# The effect of magnesium on mitotic spindle formation in *Schizosaccharomyces pombe*

**DOI:** 10.1590/1678-4685-GMB-2015-0239

**Published:** 2016-07-07

**Authors:** Gulsen Uz, Aysegul Topal Sarikaya

**Affiliations:** 1 Department of Molecular Biology and Genetics, Faculty of Arts & Sciences, Istanbul Yeni Yuzyil University, Istanbul, Turkey; 2 Faculty of Medicine, Istanbul Yeni Yuzyil University, Istanbul, Turkey

**Keywords:** magnesium, microtubule, mitosis, mitotic spindle, Schizosaccharomyces pombe

## Abstract

Magnesium (Mg^2+^), an essential ion for cells and biological systems, is involved in a variety of cellular processes, including the formation and breakdown of microtubules. The results of a previous investigation suggested that as cells grow the intracellular Mg^2+^ concentration falls, thereby stimulating formation of the mitotic spindle. In the present work, we used a Mg^2+^-deficient *Schizosaccharomyces pombe* strain GA2, in which two essential membrane Mg^2+^ transporter genes (homologs of *ALR1* and *ALR2* in *Saccharomyces cerevisae*) were deleted, and its parental strain Sp292, to examine the extent to which low Mg^2+^ concentrations can affect mitotic spindle formation. The two *S. pombe* strains were transformed with a plasmid carrying a GFP-α2-tubulin construct to fluorescently label microtubules. Using the free Mg^2+^-specific fluorescent probe mag-fura-2, we confirmed that intracellular free Mg^2+^ levels were lower in GA2 than in the parental strain. Defects in interphase microtubule organization, a lower percentage of mitotic spindle formation and a reduced mitotic index were also observed in the GA2 strain. Although there was interphase microtubule polymerization, the lower level of mitotic spindle formation in the Mg^2+^-deficient strain suggested a greater requirement for Mg^2+^ in this phenomenon than previously thought.

## Introduction

Magnesium (Mg^2+^) is the most abundant intracellular divalent cation in cells. Most of the Mg^2+^ in cells is bound to nucleotides, proteins and phospholipids; only a small portion of the total Mg^2+^ (~0.3-1.2 mM) is free in cells (Romani and Scarpa, 2000). Magnesium plays a vital role as a cofactor for hundreds of enzymes and as a stabilizer for nucleic acids and biomembranes. The most studied function of Mg^2+^ is its complexation with ATP^4-^ (Mg-ATP^2-^). In this complex, Mg^2+^ facilitates transphosphorylation reactions that are crucial for signal transduction. Cellular proliferation, which is a complex cellular process, starts with receptor-mediated mitotic signals and continues via phosphorylation-based signal transduction that leads to cell cycle progression and subsequent mitosis. Magnesium (both ionized and bound forms) is involved in nearly every step of cellular proliferation, from its initiation via receptor-mediated mitotic signals to DNA replication, cytoskeletal re-arrangements, the formation of the mitotic spindle and cytokinesis (Wolf and Trapani, 2008).

In animals, Mg^2+^ is important for muscle contraction, nerve impulse conduction and bone formation. Many diseases, such as hypertension, cardiovascular disease, tetany, seizures, depression, type 2 diabetes, metabolic syndrome, and osteoporosis, are associated with an impaired Mg^2+^ balance (Musso, 2009). Cancer is also associated with an impaired Mg^2+^ balance. Several animal model studies demonstrated that Mg^2+^ has a protective effect during the early phases of some types of cancer that are induced by chemicals (Kasprzak *et al.*, 1994; Mori *et al.*, 1997; Patiroglu *et al.*, 1997). Epidemiological studies have also demonstrated an association between Mg^2+^ deficiency and some types of human cancers (Chiu *et al.*, 2010). A study in mice demonstrated that Mg^2+^ deficiency prevents primary tumor growth but increases metastases (Nasulewicz *et al.*, 2004). Magnesium homeostasis is therefore vital to cells and is tightly controlled.

The intracellular Mg^2+^ concentration is regulated by the uptake of Mg^2+^ across the cell membrane, the efflux of Mg^2+^ from the cell and the storage of Mg^2+^ within organelles, such as mitochondria, the endoplasmic reticulum and the nucleus (Lim *et al.*, 2011). A negative membrane potential inside the cell generates a driving force for Mg^2+^ uptake across the cell membrane via ion channels or carriers (Nishizawa *et al.*, 2007). The CorA channel protein family is the major group of Mg^2+^ transporters in prokaryotes and eukaryotes (Moomaw and Maguire, 2008). The prokaryotic CorA protein is responsible for the transport of Mg^2+^, as well as Co^2+^ and Ni^2+^ ions. The eukaryotic CorA protein family includes the yeast Alr1 and Alr2 proteins, which are located in the plasma membrane and responsible for the transport of Mg^2+^ and other cations (Co^2+^, Mn^2+^, Ni^2+^, Cu^2+^, Ca^2+^, La^3+^ and Al^3+^). The other eukaryotic CorA protein family members are Mrs2 and Lpe10, which are located in the mitochondrial membrane and responsible for Mg^2+^ transport (Bui *et al.*, 1999). Unlike the other CorA homologs, the expression of the yeast Alr1 and Alr2 proteins was reported to be regulated by the Mg^2+^ supply; however the mechanism that underlies this regulation remains unknown (Graschopf *et al.*, 2001).

The role of Mg^2+^ in cellular proliferation and the cell cycle has been studied in yeasts. Yeast cells grown in low Mg^2+^ medium showed growth arrest, an increase in the percentage of cells in G0/G1 and G2/M and a decrease in the percentage of cells in S phase. In addition, the intracellular total Mg^2+^ concentration decreased as the cells grew (Rubin, 1975; Walker and Duffus, 1980). The decrease in Mg^2+^ concentration was postulated to continue to a level that permitted tubulin polymerization and formation of the mitotic spindle, then shortly before cell division, a rapid Mg^2+^ influx occurred that raised the intracellular Mg^2+^ to a concentration that caused breakdown of the mitotic spindle (Walker and Duffus, 1980). However, additional studies on the mechanism of Mg^2+^ uptake and its effect on tubulin in yeast are needed.

In this study, we investigated the effect of Mg^2+^ on mitotic spindle formation in the model organism *Schizosaccharomyces pombe*. To determine how the mitotic spindle is affected by a Mg^2+^ deficiency and availability, we used a mutant strain of *S. pombe* in which two essential genes that are responsible for membrane Mg^2+^ transport were deleted and compared this strain with the control parental strain. We introduced a GFP-tagged α2 tubulin expression plasmid (pDQ105) into *S. pombe* strains to visualize microtubules and we used the mag-fura-2 AM probe to determine the intracellular free Mg^2+^ concentration. The polymerization of mitotic microtubules was assessed in Mg^2+^-deficient *S. pombe* and in the control strain. We also examined whether the influx of Mg^2+^ influenced division in the *S. pombe* strains.

## Material and Methods

### Strains, growth media and standard methods

The *S. pombe* strains Sp292 (*leu1-32 ura4-D18 ade6*-*M210* h-), which was used as a control, and GA2 (Δ*SPAC17A2.14* Δ*SPBC27B12.12 leu1-32 ura4-D18 ade6-M216 kanr*), a Mg^2+^-deficient strain, were used. Standard YEL- and YEA-rich yeast extract media (Gutz *et al.*, 1974) were used to cultivate the Sp292 strain, and Mg^2+^-supplemented yeast extract medium (YEL containing 75 mM MgCl_2_; YEA containing 200 mM MgCl_2_) were used to cultivate the GA2 strain. The number of cells in yeast suspensions was determined by measuring the optical density at 595 nm (OD595) and comparing the results to a standard curve. Generation times and numbers were determined. The PDQ105 plasmid that carries a GFP-tagged α-tubulin was a kind gift from Dr. Da-Qiao Ding (National Bioresource Project, Yeast Genetic Resource Center, Osaka, Japan). The yeast strains were transformed with the PDQ105 plasmid using a standard method (Moreno *et al.*, 1991). For Sp292, the minimal media MMA and MML (Gutz *et al.*, 1974) supplemented with adenine and uracil (225 mg/liter) were used as selective media for the transformants. For the GA2 strain, 200 mM MgCl_2_ was added to MMA and 75 mM MgCl_2_ was added to MML, in addition to adenine and uracil.

### Determination of free intracellular magnesium (Mg^2+^) in *S. pombe* strains using ratiometric imaging

The experimental protocol was done as described by Zhang *et al.* (1997), with some modifications. Briefly, 1 mL of cell suspension from mid-log phase cultures (0.5-1×10^7^ cells/mL) of Sp292 and GA2 that expressed GFP-tagged α-tubulin was washed twice with 1 mL of Tris-HCl buffer solution (10 mM Tris-HCl, pH 7.6, containing 10 mM glucose and 135 mM NaCl), and the cells were suspended in 1 mL of Tris-HCl buffer. The loading facilitator Pluronic F-127 was added at a final concentration of 15 μmol/L and the solution was mixed. The Mg^2+^-specific, membrane permeable probe mag-fura-2 AM was added at a final concentration of 5 μmol/L in Tris-HCl buffer and the cells were incubated at 30 °C for 2 h. The cells were washed twice with Tris-HCl buffer to remove excess dye and incubated in Tris-HCl buffer for an additional 30 min to allow complete hydrolysis of the dye. To immobilize the cells prior to ratiometric imaging, the Petri dishes were coated with concanavalin A (con A; 1 mg/mL), prior to the addition of 1 mL of cell suspension containing cells loaded with mag-fura-2 AM. Ratiometric imaging was done using an Olympus 1×71 inverted epifluorescence microscope (KeyMed Ltd., United Kingdom). Mag-fura-2-loaded and GFP-tagged α-tubulin-expressing cells were identified using MetaFluor 6.2r4 software (Molecular Devices, Sunnyvale, CA, USA). Fluorescence intensities at an emission wavelength of 510 nm were recorded at 340 (200 ms) and 380 (30 ms) nm excitation wavelengths for mag-fura-2 and a 470 nm excitation wavelength for GFP. The intensities were recorded at 2-minute intervals using a CCD camera (Photometrics Coolsnap HQ, USA). Ratio values were obtained from the mag-fura-2 loaded cells. To determinate the absolute values of intracellular free magnesium ([Mg^2+^]_i_) in the cells according to the formula described by Grynkiewicz *et al.* (1985), an *in situ* calibration was done in Petri dishes containing mag-fura-2-loaded cells. MgCl_2_ was added to final concentrations of 1, 10, 30 and 60 mM (max) to determine the R_max_, and EDTA was added to concentrations of 10, 20, 30, 40, 50, 60 and 80 mM to remove all Mg^2+^ from the media and determine the R_min_. The ratio values were obtained for 10 cells of each strain, and the mean ± SEM values were calculated to determine the statistical significance of the observed differences using an unpaired *t*-test (GraphPad Software, Inc., USA).

### *In vivo* fluorescence imaging of microtubules

Sp292 cells carrying the PDQ105 plasmid (Ding *et al.*, 1998) were grown in selective minimal medium supplemented with adenine and uracil (225 mg/L). For the GA2 strain, 75 mM MgCl_2_ was added to MML medium, in addition to adenine and uracil. To observe chromosomes and microtubules simultaneously in log phase cells, Hoechst33342 was added to the culture medium (final concentration – 10 μg/mL) and incubated for 30 min at 30 °C. Cells that expressed GFP-tagged α-tubulin and stained with Hoechst33342 were transferred to Petri dishes coated with concanavalin A (1 mg/mL) and observed with an Olympus 1×71 inverted epifluorescence microscope using GFP and DAPI filters.

## Results and Discussion

### Measurement of intracellular free Mg^2+^ using mag-fura-2

Mag-fura-2 probe-loaded *S. pombe* cells were chosen to determine the intracellular free Mg^2+^ concentration using MetaFluor 6.2r4 software. The fluorescence intensity at 340 nm and 380 nm and the fluorescence ratio values (i.e., 340/380 nm) were acquired. *In situ* calibration was done to allow calculation of the intracellular free Mg^2+^ concentration according to Formula 1 described by Grynkiewicz *et al.* (1985)).

(1) $$[{\text{Mg}}^{2+}]={\text{K}}_{\text{d}}\left[\frac{{\text{F}}_{0}}{{\text{F}}_{\text{S}}}\right]\left[\frac{\text{R}-{\text{R}}_{\min}}{{\text{R}}_{\max}-\text{R}}\right]$$

where R is the ratio of the fluorescence intensity at 340:380 nm, R_min_ is the same ratio in the absence of Mg^2+^, R_max_ is the same ratio with a maximum Mg^2+^ concentration, K_D_ is the dissociation constant of the mag-fura-2/Mg^2+^ complex, and F_0_ and F_S_ are the fluorescence intensities at 380 nm in the absence of Mg^2+^ and saturated with Mg^2+^, respectively.

The R_min_, R_max_, F_0_ and F_S_ values, which are necessary for calculating the intracellular free Mg^2+^ concentration, could not be determined. For this reason, the intracellular free Mg^2+^ concentration could not be calculated. During *in situ* calibration, when the extracellular Mg^2+^ concentration was increased to 1, 10, 20 and 30 mM, the fluorescence ratio values were only slightly increased (Supplemental Figure S1). (Ratio values for the intracellular Mg^2+^ concentration were not included in the manuscript because the values were for calibration). The ratio values for increased levels of Mg^2+^ and EDTA are included as a supplemental figure) During attempts to obtain the R_min_ value, when EDTA was added to concentrations of 5, 50, 100 and 200 mM, the ratio values were only slightly decreased (Supplemental Figure S2). These findings suggested that fission yeasts have a unique transport system and that Mg^2+^ transport is tightly regulated (Zhang *et al.*, 1997).

**Figure 1:**
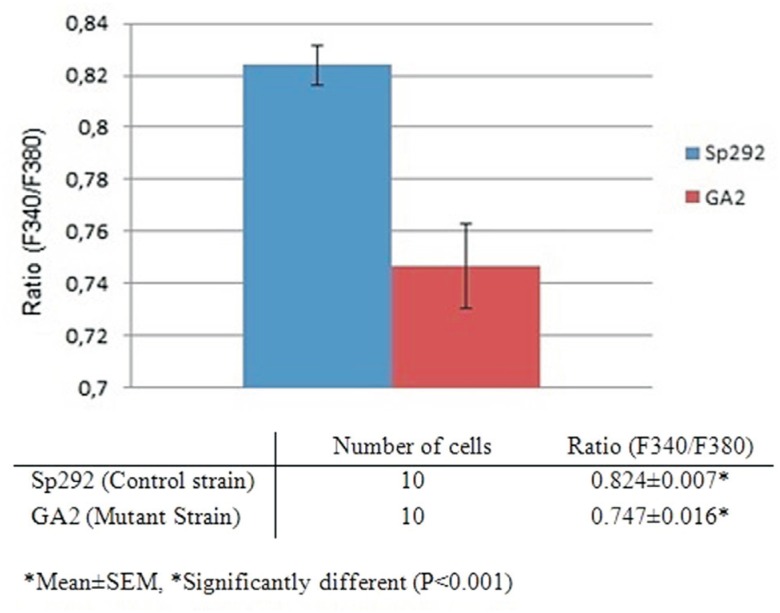
Comparison of the ratio values of the Sp292 and GA2 strains. Sp292 cells were incubated with YEL and GA2 cells were incubated with YEL supplemented with 75 mM MgCl_2_. The cells were harvested during the mid-log phase. The columns represent the mean ± SEM of at least three experiments.

The fluorescence intensity levels at 340 nm and 380 nm were reduced after 30-40 min. However, at the beginning of the experiments, the fluorescence intensity levels were constant for 10-20 min. During this time period, the ratio values, which indicate intracellular free Mg^2+^ levels, of Sp292 (the control strain) and GA2 (the Mg^2+^ transport system-deficient strain) were compared. The ratio values of GA2 cells were significantly lower than those of Sp292 cells (Figure 1).

Reductions in the fluorescence intensity at 340 nm and 380 nm suggested that the *S. pombe* cells were pumping the probe out of the cell. However, because mag-fura-2 is a ratiometric probe, the fluorescence signals of the probe/Mg^2+^ complex are independent of the probe concentration (Barreto-Chang and Dolmetsch, 2009). In view of this property of the mag-fura-2 probe, ratio values, which indicate free Mg^2+^ levels, were compared between the control and mutant strains of *S. pombe*. However, the ratio values increased over time as the fluorescence intensity decreased over time. For this reason, the cells that had the lowest ratio values were analyzed.

Dividing *S. pombe* cells have a septum during the G1-S phase of the cell cycle. Sp292 cells containing a septum (i.e., cells in the G1-S phase) were analyzed and compared with cells in the G2-M phase of the cell cycle. The ratio values of G1-S and G2-M phase cells were not significantly different (Supplemental Figure S3). (It was technically difficult to compare ratio values in G1-S and G2-M for both strains because septum formation was very low in the GA2 strain, making it hard to determine the cell cycle stage in GA2. Nevertheless, the ratio values of the G1-S and G2-M phase in the parental strain are provided in Supplemental Figure S3. The ratio values ranged from 0.8-1.0 during different phases of the cell cycle (S3). This finding suggested that differences exist between the early and late phases of G1-S or G2-M. Previous studies have also shown that intracellular free Mg^2+^ levels vary at different stages of the cell cycle (Walker and Duffus, 1980; Zhang *et al.*, 1997).

The hypothesis that a Mg^2+^ influx occurs before chromosome segregation was investigated (Walker and Duffus, 1980). To determine whether a Mg^2+^ influx occurred in dividing Sp292 cells that had a healthy Mg^2+^ transport system, ratio values were tracked in cells during cell division and at different stages of cell division. Differences in the ratio values ranged from 0.8-1.0, suggesting that a Mg^2+^ influx may have occurred in G1-S or G2-M; in contrast, the ratio values at different stages of cell division were not significantly different from each other. However, since we did not use synchronized cells in our experiments additional studies using cells synchronized in the G2 phase are needed to test this hypothesis.

### The effect of intracellular free Mg^2+^ on mitotic spindle formation

**Figure 2:**
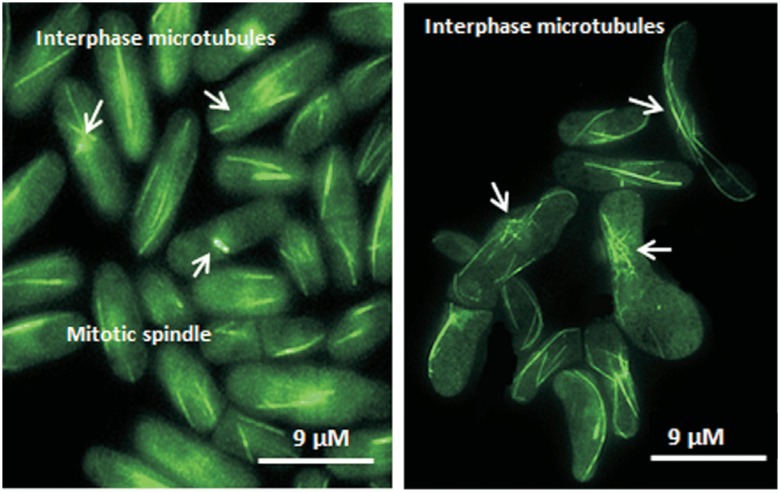
Microtubule organization in the Sp292 and GA2 strains. A. The arrows indicate healthy interphase microtubules and mitotic spindle in Sp292. B. The arrows indicate defective interphase microtubule organization in the GA2 strain.

In this study, we aimed primarily to observe fluorescently labeled free Mg^2+^ in GFP-tagged microtubule-expressing cells in different phases of the cell cycle. However, no GFP-tagged microtubules were observed in mag-fura-2 probe-loaded *S. pombe* cells. This finding indicated either that GFP-tagged microtubules are sensitive to mag-fura-2 or that mag-fura-2 had a quenching effect on GFP. Because GFP-tagged microtubules could not be observed in cells containing fluorescently labeled free Mg^2+^, a yeast strain in which the Mg^2+^ transport was deleted (GA2) was analyzed to determine how Mg^2+^ deficiency affected formation of the mitotic spindle. For this purpose, mitotic spindle formation in a Mg^2+^-deficient strain that expressed GFP-tagged α2-tubulin was compared with the control strain. In addition, the percentage of cells that generated a mitotic spindle was analyzed in both strains. Initially, we observed defective organization of the interphase microtubules in the GA2 strain (Figure 2). Since the microtubular cytoskeleton is important for normal cell morphology in fission yeast (Mata and Nurse, 1997; Beinhauer *et al.*, 1997), GA2 cells were also defective in cell morphology.

The mitotic index was lower in the Mg^2+^-deficient GA2 strain than in Sp292, as expected. Magnesium restriction can result in failure to divide in fission yeast cells (Walker and Duffus, 1980). Because Mg^2+^ is involved in mitotic signals that are mediated by DNA synthesis, the formation of the mitotic spindle and cytokinesis, it is very important to determine how a Mg^2+^ deficiency affects cell division and the mitotic index. Consequently, the Mg^2+^ transport system-deficient mutant strain GA2 is a useful model system for elucidating the relationship between Mg^2+^ and the mitotic spindle.

**Figure 3:**
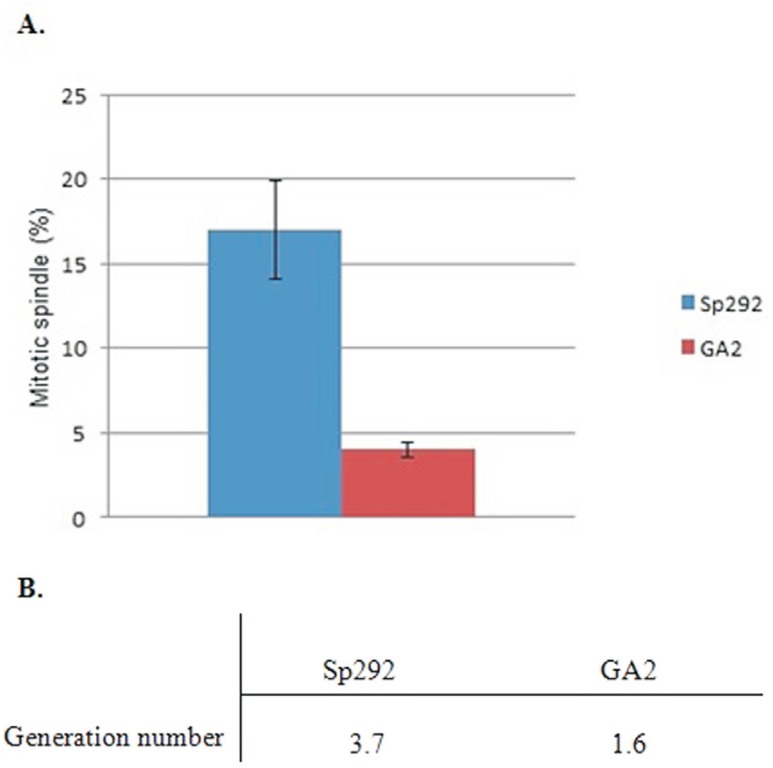
Comparison of the percentage of mitotic spindles and generation number in Sp292 and GA2 cells. A. Percentage of GA2 and Sp292 cells with a mitotic spindle during the log phase. At least 100 GFP-tagged α-tubulin-expressing cells were counted. B. Generation numbers for Sp292 and GA2.

After counting the number of mitotic spindle-carrying cells in relation to dividing cells, the percentage of cells with a mitotic spindle was estimated and compared between GA2 and Sp292 cells. The percentage of cells with a mitotic spindle was lower in GA2 cells than in Sp292 cells (Figure 3A). Generation numbers in logarithmic phase were also calculated using a standard formula (Formula 2) (Boyd, 1984). GA2 cells were estimated to divide approximately once, while Sp292 cells divided three times (Figure 3B). The reduced percentage of mitotic spindles and the number of generations in the Mg^2+^-deficient GA2 strain suggested that a Mg^2+^ deficiency reduced the mitotic spindle formation and therefore the generation number (i.e., the mitotic index was lower in GA2 cells than in Sp292 cells).

(2) $$\text{Generation number}(\text{n})=\frac{\log{\text{N}}_{2}-\log{\text{N}}_{1}}{\log_{2}}$$

where N_2_= Number of cells in culture at the end of the logarithmic phase and N_1_= Number of cells present in culture at the beginning of the logarithmic phase

**Figure 4:**
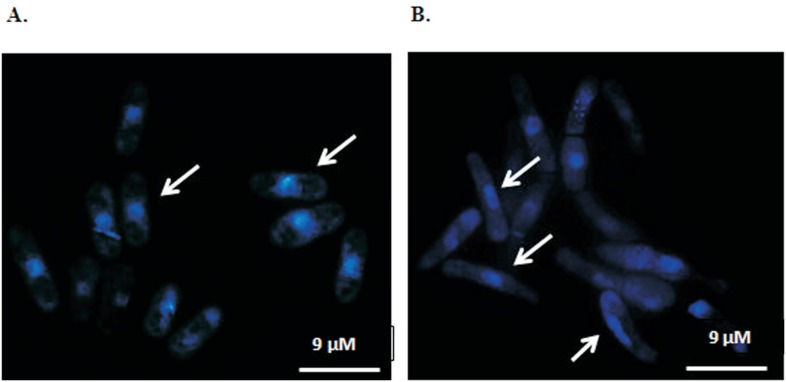
Comparison of nuclear images in Sp292 and GA2. A. Nuclear images of Sp292 and GA2. B. Images of stationary phase cells. The arrows indicate the nucleus in Sp292 and GA2 cells. The nuclei were stained with Hoechst 33342.

We also investigated whether chromosome segregation occurred in GA2 cells. Nuclear images revealed that most Mg^2+^ -deficient GA2 cells were elongated (Supplemental Table S1), had no division septum and were mononucleated (Figure 4A). In contrast, Sp292 cells were small and mononucleated, which is characteristic of fission yeast cells during the stationary phase (Figure 4B). Our findings are consistent with those of Walker and Duffus (1980) in which a Mg^2+^ deficiency was generated by removing Mg^2+^ from the growth medium. The culture medium for the Mg^2+-^transport system-deficient strain GA2 was supplemented with 75 mM MgCl_2_. This supplementation was necessary because GA2 cells cannot divide even once without Mg^2+^ supplementation. However, Mg^2+^ supplementation of GA2 cells only supports a single division and increasing the Mg^2+^ concentration to > 75 mM does not increase the generation number of GA2.

Figure 4A shows elongated GA2 cells with mononuclei; this finding suggests that chromosomal segregation did not occur due to reduced mitotic spindle formation. In contrast, our observations revealed that more Mg^2+^ was required for spindle formation than for the polymerization of interphase microtubules. However, the defective organization of interphase microtubules also demonstrated that Mg^2+^ is important for the intactness of interphase microtubules or cytoplasmic microtubules.

Previous work has shown that Mg^2+^ restriction causes most mammalian cells to be arrested first in the S phase and then in the G2/M phase of the cell cycle (Wolf and Trapani, 2008). However, the presence of elongated GA2 cells with mononuclei showed that these cells could not pass the G2/M phase of cell cycle. This finding suggests that Mg^2+^ deficiency has different effects on the cell cycle in fission yeasts. In addition, the observed cell cycle arrest at G2/M in GA2 cells may have occurred at the spindle-assembly checkpoint. However, *SPAC17A2.14*, which is a homolog of the *S. cerevisiae* Mg^2+^ transporter *ALR1*, was reported to interact with the Sad1 protein, which is a component of the microtubule-organizing center (MTOC) (Miki *et al.*, 2004). Sad1 was previously demonstrated to be essential for spindle formation and elongation (Hagan and Yanagida, 1995). Consequently, deletion of the genes that are responsible for Mg^2+^ transport may have affected many cellular pathways. Future work will seek to identify these cellular pathways by using the Mg^2+^-deficient mutant strain as a model system.

## Supplementary Material

The following online material is available for this article:

Figure S1 - Ratio values for intracellular Mg^2+^ after increasing extracellular Mg^2+^.

Figure S2 - Ratio values for intracellular Mg^2+^ after EDTA addition.

Figure S3 - Ratio values for the G1-S and G2-M phase.

Table S1 - Comparison of cell length (μm) between Sp292 and GA2 cells

## References

[B1] Barreto-Chang OL, Dolmetsch RE (2009). Calcium imaging of cortical neurons using Fura-2 AM. J Vis Exp.

[B2] Beinhauer JD, Hagan IM, Hegemann JH, Fleig U (1997). Mal3, the fission yeast homolog of the human APC-interacting protein
EB-1 is required for microtubule integrity and the maintenance of cell
form. J Cell Biol.

[B3] Boyd RF (1984). General Microbiology.

[B4] Bui DM, Gregan J, Jarosch E, Ragnini A, Schweyen RJ (1999). The bacterial magnesium transporter CorA can functionally substitute
for its putative homologue Mrs2p in the yeast inner mitochondrial
membrane. J Biol Chem.

[B5] Chiu HF, Tsai SS, Wu TN, Yang CY (2010). Colon cancer and the content of nitrate and magnesium in drinking
water. Mag Res.

[B6] Ding DQ, Chikashige Y, Haraguchi T, Hiraoka Y (1998). Oscillatory nuclear movement in fission yeast meiotic prophase is
driven by astral microtubules, as revealed by continuous observation of
chromosomes and microtubules in living cells. J Cell Sci.

[B7] Graschopf A, Stadler JA, Hoellerer MK, Eder S, Sieghardt M, Kohlwein SD, Schweyen RJ (2001). The yeast plasma membrane protein Alr1 controls Mg^2+^
homeostasis and is subject to Mg^2+^ dependent control of its synthesis
and degradation. J Biol Chem.

[B8] Grynkiewicz G, Poenie M, Tsien RY (1985). A new generation of Ca^2+^ indicators with greatly improved
fluorescence properties. J Biol Chem.

[B9] Gutz H, Heslot H, Leupold U, Loprieno N, King RC (1974). Schizosaccharomyces pombe. Handbook of Genetics.

[B10] Hagan I, Yanagida M (1995). The product of spindle formation gene *sad1*+
associates with the fission yeast spindle pole body and is essential for
viability. J Cell Biol.

[B11] Kasprzak KS, Diwan BA, Rice JM (1994). Iron accelerates while magnesium inhibits nickel-induced
carcinogenesis in the rat kidney. Toxicology.

[B12] Lim PH, Pisat NP, Gadhia N, Pandey A, Donovan FX, Stein L, Salt DE, Eide DJ, Macdiarmid CW (2011). Regulation of Alr1 Mg transporter activity by intracellular
magnesium. PLoS One.

[B13] Mata J, Nurse P (1997). Tea1 and the microtubular cytoskeleton are important for generating
global spatial order within the fission yeast cell. Cell.

[B14] Miki F, Kurabayashi A, Tange Y, Okazaki K, Shimanuki M, Niwa O (2004). Two-hybrid search for proteins that interact with Sad1 and Kms1, two
membrane-bound components of the spindle pole body in fission
yeast. Mol Genet Genomics.

[B15] Moomaw AS, Maguire ME (2008). The unique nature of Mg^2+^ channels. Physiology (Bethesda).

[B16] Moreno S, Klar A, Nurse P (1991). Molecular genetic analysis of *Schizosaccharomyces
pombe*. Methods Enzymol.

[B17] Mori H, Tanaka T, Sugie S, Yoshimi N, Kawamori T, Hirose Y, Ohnishi M (1997). Chemoprevention by naturally occurring and synthetic agents in oral,
liver and large bowel carcinogenesis. J Cell Biochem.

[B18] Musso CG (2009). Magnesium metabolism in health and disease. Int Urol Nephrol.

[B19] Nasulewicz A, Wietrzyk J, Wolf FI, Dzikir AS, Madej J, Maier JA, Rayssiguier Y, Mazur A, Opolski A (2004). Magnesium deficiency inhibits primary tumor growth but favors
metastasis in mice. Biochim Biophys Acta - Mol Basis Dis.

[B20] Nishizawa Y, Morii H, Durlach J (2007). New Perspectives in Magnesium Research Nutrition and Health.

[B21] Patiroglu T, Sahin G, Kontas O, Uzüm K, Saraymen R (1997). Protective effect of magnesium supplementation on experimental
3-methyl cholanthrene-induced fibrosarcoma and changes in tissue magnesium
distribution during carcinogenesis in rats. Biol Trace Elem Res.

[B22] Romani AM, Scarpa A (2000). Regulation of cellular magnesium. Front Biosci.

[B23] Rubin H (1975). Central role for magnesium in coordinate control of metabolism and
growth in animal cells. Proc Natl Acad Sci USA.

[B24] Walker GM, Duffus JH (1980). Magnesium ions and the control of the cell cycle in
yeast. J Cell Sci.

[B25] Wolf FI, Trapani V (2008). Cell (patho)physiology of magnesium. Clin Sci.

[B26] Zhang A, Cheng TP, Wu XY, Altura BT, Altura BM (1997). Extracellular Mg^2+^ regulates intracellular Mg^2+^
and its subcellular compartmentation in fission yeast, *Schizosaccharomyces
pombe*. Cell Mol Life Sci.

