# Supplementary material for: The effect of magnesium on mitotic spindle formation in Schizosaccharomyces pombe
Source: Genet Mol Biol. 2016 Jul 7;39(3):459–64. doi: 10.1590/1678-4685-GMB-2015-0239 (PMC5004833; doi:10.1590/1678-4685-GMB-2015-0239)
Supplement: Supplementary file 1 [file 1415-4757-gmb-1678-4685-GMB-2015-0239-Suppl01.pdf]

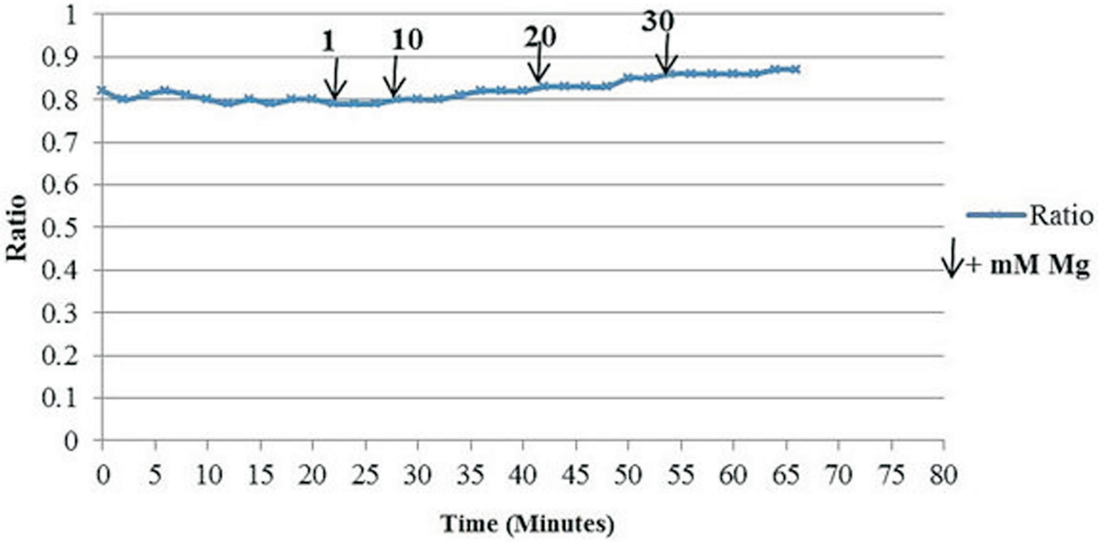

Figure S1 - Ratio values showing the intracellular Mg^{2+} concentration when the extracellular Mg^{2+} concentration was increased to 1, 10, 20 and 30 mM (at arrows).
