# Supplementary material for: The effect of magnesium on mitotic spindle formation in Schizosaccharomyces pombe
Source: Genet Mol Biol. 2016 Jul 7;39(3):459–64. doi: 10.1590/1678-4685-GMB-2015-0239 (PMC5004833; doi:10.1590/1678-4685-GMB-2015-0239)
Supplement: Supplementary file 2 [file 1415-4757-gmb-1678-4685-GMB-2015-0239-Suppl02.pdf]

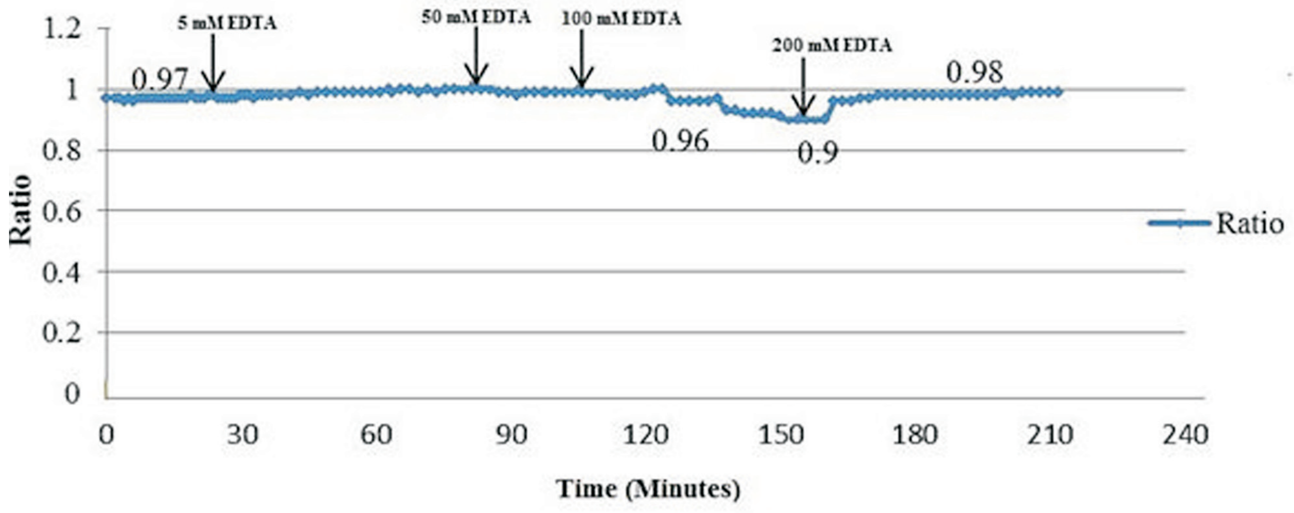

Figure S2 - Ratio values showing the intracellular Mg^{2+} concentration when EDTA was added extracellularly to concentrations of 5, 50, 100 and 200 mM.
