# Supplementary figures and images for: The effect of magnesium on mitotic spindle formation in Schizosaccharomyces pombe
Source: Genet Mol Biol. 2016 Jul 7;39(3):459–64. doi: 10.1590/1678-4685-GMB-2015-0239 (PMC5004833; doi:10.1590/1678-4685-GMB-2015-0239)

**A**

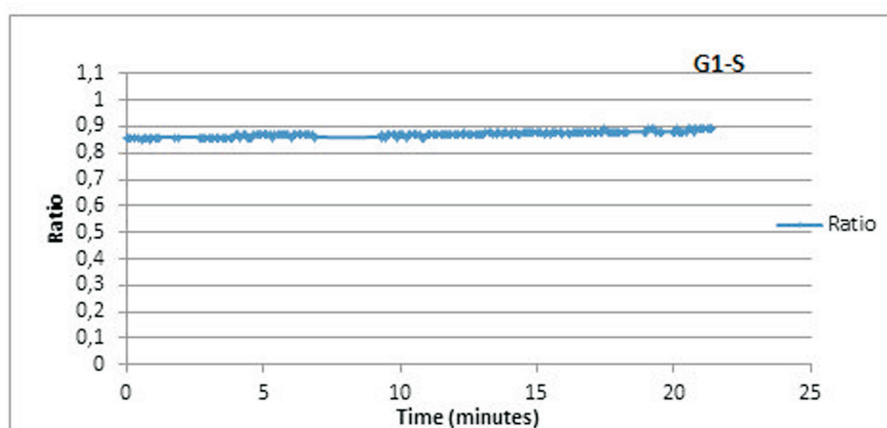

**B**

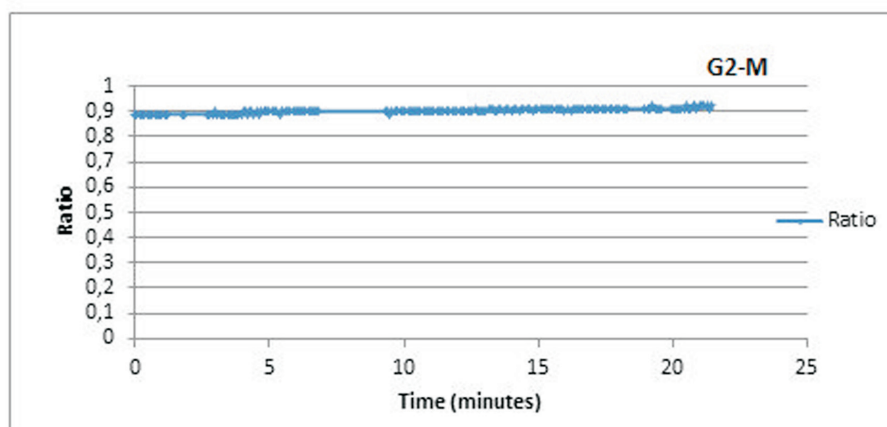

**Figure S3** - Ratio values for the G1-S phase (A) and G2-M phase (B) in Sp292 cells.

Supplement: Supplementary file 3 [file 1415-4757-gmb-1678-4685-GMB-2015-0239-Suppl03.pdf]
