# Supplementary material for: The effect of magnesium on mitotic spindle formation in Schizosaccharomyces pombe
Source: Genet Mol Biol. 2016 Jul 7;39(3):459–64. doi: 10.1590/1678-4685-GMB-2015-0239 (PMC5004833; doi:10.1590/1678-4685-GMB-2015-0239)
Supplement: Supplementary file 4 [file 1415-4757-gmb-1678-4685-GMB-2015-0239-Suppl04.pdf]

Supplemental Data

Table S1: Comparison of cell length (µm) between Sp292 and GA2 cells

**GA2 (cell number)**	**Cell Length (µm)**
1	5.976
2	7.450
3	11.564
4	16.721
5	11.914
6	7.204
**Average**	**10.138**
**SD**	**3.690**
**Sp292(cell number)**	**Cell Length (µm)**
1	4.379
2	5.715
3	6.552
4	3.534
5	4.413
6	5.321
7	5.820
8	5.746
**Average**	**5.185**
**SD**	**0.925**

Cell length for **GA2** strain: 10.138 ±3.69 µm and for **SP292** strain: 5.185 ± 0.925 µm. ImageJ program was used for cell length measurements.
